# Prevalence, characteristics, and emotional effects of school bullying among Grade 5–6 students in Hat Yai, southern Thailand: a cross-sectional study

**DOI:** 10.1186/s40359-026-05346-z

**Published:** 2026-08-10

**Authors:** Pim Srimekarat, Napakkawat Buathong, Ruthaychonnee Sittichai, Pitchayanont Ngamchaliew

**Affiliations:** 1 Department of Family and Preventive Medicine, Faculty of Medicine, Prince of Songkla University, Hat Yai, Songkhla 90110 Thailand; 2 Social Development Administration Program, Faculty of Humanities and Social Sciences, Prince of Songkla University, Pattani, 94000 Thailand

**Keywords:** Bullying, Cyberbullying, Bully-victim, Primary school, Parenting style, Depression, PHQ-A, Thailand, Mental health

## Abstract

**Background:**

School bullying is a common form of youth violence and an adverse experience with well-established effects on mental health and development. Thailand reports one of the highest prevalence rates globally. However, evidence concerning bullying in primary school populations, particularly in the southern region of Thailand, remains limited. This study examined the prevalence and characteristics of traditional bullying and cyberbullying and identified the factors associated with bullying roles among students in Grades 5 and 6 in Hat Yai, southern Thailand.

**Methods:**

We conducted a school-based, cross-sectional survey in one public and one private primary school. A total of 291 students participated. Data were collected using self-administered questionnaires, including the Thai versions of the Revised Olweus Bully/Victim Questionnaire, the Cyber-Aggression Perpetration/Victimization Scale, and the Patient Health Questionnaire for Adolescents. Students were classified as victims, bullies, bully-victims, and those not involved. Associated factors were identified using multinomial logistic regression and reported using relative-risk ratios (RRRs) with 95% confidence intervals (CIs).

**Results:**

Overall, 78.7% of the participants were involved in bullying, with bully-victims constituting the largest group (44.7%), followed by victims (30.2%) and bullies (3.8%); a sensitivity analysis restricted to the two global R-OBVQ items yielded an involvement prevalence of 59.1%. Verbal bullying was the most common form of bullying, and incidents frequently occurred in unsupervised areas in the school. Moderate-to-severe depressive symptoms were strongly associated with bullying involvement, particularly with being a bully-victim. In adjusted analyses, attending a private school (RRR 4.48, 95% CI 1.97–10.22) and authoritarian parenting (RRR 4.20, 95% CI 1.24–14.21) were independently associated with a higher relative risk of being a bully-victim, whereas shorter-than-average stature was associated with a lower relative risk (RRR 0.27, 95% CI 0.08–0.93). Cyberbullying was uncommon (5.2%) and was not significantly associated with bullying roles.

**Conclusions:**

Bullying involvement is highly prevalent among primary school students in southern Thailand, with bully-victims constituting the largest and the most vulnerable group in terms of mental health. These findings highlight the need to integrate mental health screening, parenting-focused interventions, and improved supervision within school environments to prevent school bullying.

## Background

Bullying is defined as repeated aggressive behaviour from one individual to another, where the relationship often involves a real or perceived power imbalance [1]. Traditional bullying can involve physical harm, verbal abuse, relational aggression, or damage to one’s property. Meanwhile, cyberbullying refers to aggression directed through digital and electronic-communication platforms [2, 3].

Schools are the most common setting where bullying occurs. Recognised as one of the most prevalent forms of youth violence, bullying disproportionately affects school-aged populations worldwide. Moreover, its consequences extend well beyond the school years. Bullying involvement has been consistently linked to depression, anxiety, loneliness, suicidal ideation, poor academic performance, and low quality of life in adolescence and adulthood [4–16]. Of particular concern is the bully-victim subgroup (i.e., children who are involved both as victims and as bullies), which tends to carry the highest mental health burden across bullying roles [4, 14, 15].

According to UNESCO, approximately 32% of students aged 9–15 worldwide have experienced bullying. However, the situation appears markedly worse in Thailand. A 2020 national report described that approximately 91.8% of Thai students aged 10–15 had experienced some form of bullying, documenting one of the highest rates worldwide [17–19]. Moreover, cultural norms in Thailand, such as framing teasing and harassment as playful interactions, may delay recognition and intervention and further exacerbate bullying [20].

Despite these concerning statistics, evidence remains limited on bullying and bullying involvement among primary school students in southern Thailand. Additionally, research remains scant on how depressive symptoms, parenting environment, and school settings relate to the different roles played in bullying. A clearer understanding of role-specific correlates is essential for designing culturally appropriate, mental health-informed prevention strategies. We therefore (i) estimated the prevalence of involvement in traditional bullying and cyberbullying; (ii) determined the characteristics of traditional bullying and cyberbullying; and (iii) identified demographic, family, body size-related, and school-related factors associated with bullying roles among students in Grades 5 and 6 in Hat Yai, Songkhla, Thailand.

## Methods

### Study design and setting

We conducted a school-based cross-sectional study in Hat Yai District, Songkhla Province, southern Thailand, between August 2024 and January 2025. We selected one large public and one large private primary school to capture the major types of schools serving the urban Hat Yai population.

### Participants and sample size

Students enrolled in Grades 5 or 6 at the participating schools who could read and communicate in Thai were eligible to participate. All students studying in Grades 5 and 6 at the two schools were invited to participate. Students whose parents did not return signed informed-consent forms and students who were absent on the day of data collection were excluded.

The required sample size was estimated using a single-proportion formula (expected prevalence of 50%, 5% precision, 95% confidence, and 10% anticipated non-response) and a design effect of 2 for classroom clustering. As a result, the required sample size was 426. We received parental consent and student assent for a total of 291 students, who comprised the final sample. The public school had 102 eligible Grade 5 and 6 students (45 in Grade 5 and 57 in Grade 6), of whom 76 (74.5%) participated. The private school had 327 eligible students (146 in Grade 5 and 181 in Grade 6), of whom 215 (65.7%) participated. The overall participation rate was therefore 291/429 (67.8%). These denominators are the Grade 5 and 6 enrolment figures provided by each participating school. This shortfall in the sample, mainly due to student or parental refusal, is a potential source of participation bias, which we address in the Limitations.

### Measures

Data were collected using a self-administered, paper-based questionnaire comprising four sections. The first section consisted of items on demographic, family, physical, and school-related characteristics, which were compiled by the investigators. In the remaining sections, school bullying, cyber aggression, and depressive symptoms were assessed using previously published and validated instruments administered in their established Thai-language versions. No scale was newly developed for this study.

### Demographic, family, body size-related, and school-related characteristics

The first section captured age (in years), sex (male or female), gender identity (boy, girl, or other), religion (Buddhism, Islam, or Christianity), grade (5 or 6), type of school (public or private), grade-point average (GPA; range 0–4.0) for the most recent term, height and weight, both reported by the student rather than measured, and perceived parenting style. Sex–gender alignment was coded as “yes” when the reported gender identity corresponded to the student’s sex and “no” otherwise. The self-reported height and weight were converted into two categorical variables using the Thai national growth reference for children aged 6 to 19 years [21]: height-for-age (Shorter than average, average, or Taller than average) and weight-for-height (Thinner than average, well proportioned, or Larger than average). The original reference categories were collapsed into these three levels for analysis. Perceived parenting style was assessed using a single investigator-compiled item based on Baumrind’s four-category typology [22]. The item presented four brief descriptions of parenting behaviour, and each student selected the one that best described their main caregiver: authoritative (warm and responsive, with clear rules that are explained), authoritarian (strict control and expected obedience, with limited warmth), permissive (warm, but with few rules or demands), and uninvolved/neglectful (little guidance, warmth, or emotional engagement). Responses were coded as four mutually exclusive categories, with authoritative parenting used as the reference category in the regression analysis; because they were uncommon, the permissive and uninvolved/neglectful categories were combined in the multivariable analysis.

### Revised Olweus Bully/Victim Questionnaire (R-OBVQ), Thai version

The R-OBVQ is the most widely used self-report instrument for school bullying [23, 24]. It opens with a standard definition of bullying. It then asks two global items, on being bullied and on bullying others during the previous two months. Each item is rated on a five-point frequency scale (“it has not happened”, “only once or twice”, “two or three times a month”, “about once a week”, and “several times a week”). Further items assess specific forms of bullying (physical; verbal, including name-calling, threats, and racial and sexual comments; relational, including social exclusion and rumour spreading; and damage to property), together with the location and duration of bullying, the number, sex, and class of perpetrators, disclosure to others, and the perceived responses of teachers, guardians, and bystanders. Students were classified using the standard cutoff of “two or three times a month” or more. Victimisation was defined as reaching this cutoff on the global victimisation item or on any of the nine specific victimisation items (items 4–13), and perpetration as reaching it on the global perpetration item or on any of the nine specific perpetration items (items 24–33). Students were then assigned to four mutually exclusive roles: not involved, victim (met the victimisation criterion only), bully (met the perpetration criterion only), and bully-victim (met both criteria). This form-inclusive rule favours sensitivity over specificity; a sensitivity analysis restricted to the two global items is reported in the Results.

### Cyber-Aggression Perpetration/Victimization Scale (CAPVS), Thai version

The CAPVS comprises parallel perpetration and victimisation subscales capturing aggression carried out or experienced through digital channels, including text messages, social media, online gaming, and image sharing [25]. The validated Thai version was administered [26]. Each subscale comprises nine items rated from 0 (never) to 4, giving a subscale score of 0 to 36. Students scoring 19 or above on the perpetration subscale were classified as cyber bullies and those scoring 19 or above on the victimisation subscale as cyber victims; students who met both criteria were classified as cyber bully-victims, and all others as not involved. In this study, the terms “cyberbullying” and “cyber aggression” are used interchangeably, with cyber aggression denoting the construct operationalised by the CAPVS.

### Patient Health Questionnaire for Adolescents (PHQ-A)

Depressive symptoms over the past two weeks were measured using the validated Thai version of the PHQ-A, a nine-item self-report instrument for adolescents [27, 28]. Each item is rated from 0 (“not at all”) to 3 (“nearly every day”), giving a total score of 0–27. Total scores were categorised as no symptoms (0–4), mild symptoms (5–9), or moderate-to-severe symptoms (≥ 10), in line with established cutoffs for adolescent populations. Students whose responses indicated marked depressive symptoms, self-harm, or suicidal ideation were privately referred for clinical assessment (see Ethical considerations).

### Procedure

Research staff briefed teachers and distributed parental consent forms approximately one week before data collection. Eligible students whose parents had consented received a private explanation of the study and provided written assent. Questionnaires were completed in classrooms during a single session of approximately 45 min under the supervision of trained research assistants and without teachers present, to minimise social-desirability bias.

### Statistical analysis

Categorical variables were presented using frequencies and percentages, whereas continuous variables were presented using medians and interquartile ranges (IQRs). Differences across the four bullying roles were tested using chi-squared tests, Fisher’s exact tests (when expected cell counts were < 5), or the Kruskal–Wallis test, as appropriate. The characteristics of bullying summarised in Table 3 were compared between the public and the private school using chi-squared tests or Fisher’s exact tests applied to the categories displayed. Independent factors associated with each bullying role (victim, bully, bully-victim) were examined using multinomial logistic regression with “not involved” as the reference category. Candidate variables were those of conceptual interest or with *p* < 0.20 in bivariate analysis; the final model included sex, age, sex–gender alignment, school type, perceived parenting style, height-for-age, and weight-for-height. Grade-point average was not entered into the multivariable model because it showed no association with bullying role in bivariate analysis and its inclusion did not improve model fit; it is reported as a descriptive characteristic only. Sex was retained regardless of bivariate significance because of its well-documented association with bullying involvement. Religion was not entered into the model because 92.8% of the participants were Buddhist; the resulting sparse non-Buddhist cells would yield unstable estimates and preclude meaningful comparison. Results were reported as relative-risk ratios (RRRs) with 95% confidence intervals (CIs). Overall model performance was assessed using a likelihood-ratio test against the null model, McFadden’s pseudo-R², and the Akaike information criterion (AIC). Chi-squared tests of 2 × 2 tables included a continuity correction, and Fisher’s exact p-values were computed exactly rather than by simulation. Two-sided *p* < 0.05 was considered statistically significant.

The PHQ-A score was not entered as a predictor in the regression analysis because depressive symptoms are conceptually an outcome or mediator of bullying involvement rather than a determinant. Entering the PHQ-A score as an explanatory variable would risk reverse causation and over-adjustment by conditioning on an intermediate variable. All analyses were performed using R (version 4.1.1; R Foundation for Statistical Computing, Vienna, Austria).

### Ethical considerations

The study protocol was approved by the Human Research Ethics Committee of the Faculty of Medicine, Prince of Songkla University (approval number: REC.67-079-9-4). It adhered to the principles outlined in the Declaration of Helsinki. Written informed consent was obtained from the parent or legal guardian of each participant prior to data collection. Furthermore, written assent was obtained from each participant in a private setting after they had sufficient time to ask questions. Students whose PHQ-A responses indicated marked depressive symptoms, self-harm, or suicidal ideation were privately referred, with their consent, for assessment by a physician, and their guardians or homeroom teachers were informed and advised to seek further evaluation.

## Results

### Participant characteristics

A total of 291 students completed the questionnaire. Among them, 76 students (26.1%) studied in the public school, 215 students (73.9%) studied in the private school, 113 students (38.8%) were in Grade 5, and 175 students (60.1%) were in Grade 6 (grade was missing or incorrectly recorded for three students). Public school participants comprised 38 males and 38 females, while private school participants comprised 91 males and 121 females (sex was not recorded for three students). The median age was 11.2 years (IQR 10.9–11.8). Most participants were female (54.6%), identified as girls or boys (94.8%), reported alignment between their sex and gender (92.1%), and were Buddhists (92.8%). The median GPA was 3.7 (IQR 3.0–4.0). Demographic and body size-related characteristics did not differ significantly across bullying roles (Table 1).

**Table 1:** Demographic and body-size characteristics by bullying role

Characteristics	Total (*N* = 291)	Victim (*n* = 88; 30.2%)	Bully (*n* = 11; 3.8%)	Bully-victim (*n* = 130; 44.7%)	Not involved (*n* = 62; 21.3%)	*p*-value
Sex						0.245 F
Male	129 (44.3%)	36 (27.9%)	2 (1.6%)	63 (48.8%)	28 (21.7%)	
Female	159 (54.6%)	50 (31.4%)	9 (5.7%)	67 (42.1%)	33 (20.8%)	
Gender identity						0.351 F
Boy	126 (43.3%)	36 (28.6%)	3 (2.4%)	60 (47.6%)	27 (21.4%)	
Girl	150 (51.5%)	49 (32.7%)	7 (4.7%)	62 (41.3%)	32 (21.3%)	
Other	12 (4.1%)	1 (8.3%)	1 (8.3%)	8 (66.7%)	2 (16.7%)	
Sex–gender alignment						0.338 F
Yes	268 (92.1%)	82 (30.6%)	9 (3.4%)	119 (44.4%)	58 (21.6%)	
No	21 (7.2%)	5 (23.8%)	2 (9.5%)	11 (52.4%)	3 (14.3%)	
Age, years, median (IQR)	11.2 (10.9, 11.8)	11.1 (10.5, 11.7)	11.1 (10.7, 11.5)	11.2 (10.9, 11.8)	11.4 (11.0, 11.9)	0.244 K
GPA, median (IQR)	3.7 (3.0, 4.0)	3.6 (3.0, 4.0)	3.9 (3.8, 4.0)	3.6 (3.3, 4.0)	3.6 (2.8, 4.0)	0.392 K
Grade						0.402 F
Fifth	113 (38.8%)	39 (34.5%)	6 (5.3%)	47 (41.6%)	21 (18.6%)	
Sixth	175 (60.1%)	49 (28.0%)	5 (2.9%)	82 (46.9%)	39 (22.3%)	
Height for age						0.062 F
Taller than average	34 (11.7%)	6 (17.6%)	1 (2.9%)	21 (61.8%)	6 (17.6%)	
Average	228 (78.4%)	73 (32.0%)	10 (4.4%)	100 (43.9%)	45 (19.7%)	
Shorter than average	22 (7.6%)	8 (36.4%)	0 (0.0%)	5 (22.7%)	9 (40.9%)	
Weight for height						0.192 F
Larger than average	51 (17.5%)	18 (35.3%)	0 (0.0%)	26 (51.0%)	7 (13.7%)	
Well proportioned	182 (62.5%)	49 (26.9%)	9 (4.9%)	82 (45.1%)	42 (23.1%)	
Thinner than average	46 (15.8%)	19 (41.3%)	2 (4.3%)	16 (34.8%)	9 (19.6%)	
Religion						0.133 F
Buddhism	270 (92.8%)	78 (28.9%)	10 (3.7%)	124 (45.9%)	58 (21.5%)	
Islam	17 (5.8%)	9 (52.9%)	0 (0.0%)	5 (29.4%)	3 (17.6%)	
Christianity	3 (1.0%)	1 (33.3%)	1 (33.3%)	1 (33.3%)	0 (0.0%)	

Data are n (%) unless otherwise stated. Percentages in the Total column are column percentages of all 291 students; percentages in the Victim, Bully, Bully-victim, and Not involved columns are row percentages within each category. Numbers do not always sum to 291 because of missing responses

*F* Fisher’s exact test, *K* Kruskal–Wallis test

### Bullying-role distribution and bivariate associations

Of the 291 students, 130 (44.7%) were classified as bully-victims, 88 (30.2%) as victims, 11 (3.8%) as bullies, and 62 (21.3%) were not involved; overall, 229 students (78.7%) were involved in bullying in some role (Table 2). In a sensitivity analysis restricted to the two global R-OBVQ items, 74 students (25.4%) were classified as bully-victims, 69 (23.7%) as victims, 29 (10.0%) as bullies, and 119 (40.9%) as not involved, giving an involvement prevalence of 59.1%.

**Table 2:** Family, cyber-aggression, depressive-symptom, and school characteristics by bullying role

Characteristics	Total (*N* = 291)	Victim (*n* = 88; 30.2%)	Bully (*n* = 11; 3.8%)	Bully-victim (*n* = 130; 44.7%)	Not involved (*n* = 62; 21.3%)	*p*-value
Parenting style						0.099 F
Authoritative	216 (74.2%)	66 (30.6%)	7 (3.2%)	94 (43.5%)	49 (22.7%)	
Authoritarian	42 (14.4%)	16 (38.1%)	1 (2.4%)	21 (50.0%)	4 (9.5%)	
Permissive	17 (5.8%)	2 (11.8%)	3 (17.6%)	8 (47.1%)	4 (23.5%)	
Uninvolved/neglectful	9 (3.1%)	2 (22.2%)	0 (0.0%)	6 (66.7%)	1 (11.1%)	
Cyber-aggression role (CAPVS)						0.318 F
Victim	13 (4.5%)	4 (30.8%)	0 (0.0%)	9 (69.2%)	0 (0.0%)	
Bully	1 (0.3%)	0 (0.0%)	0 (0.0%)	1 (100.0%)	0 (0.0%)	
Bully-victim	1 (0.3%)	0 (0.0%)	0 (0.0%)	1 (100.0%)	0 (0.0%)	
Not involved	274 (94.2%)	82 (29.9%)	11 (4.0%)	119 (43.4%)	62 (22.6%)	
Depressive symptoms (PHQ-A)						< 0.001 F
None (score 0–4)	72 (24.7%)	17 (23.6%)	5 (6.9%)	19 (26.4%)	31 (43.1%)	
Mild (score 5–9)	90 (30.9%)	34 (37.8%)	2 (2.2%)	30 (33.3%)	24 (26.7%)	
Moderate to severe (score ≥ 10)	127 (43.6%)	37 (29.1%)	4 (3.1%)	79 (62.2%)	7 (5.5%)	
School type						0.003 F
Public school	76 (26.1%)	27 (35.5%)	4 (5.3%)	21 (27.6%)	24 (31.6%)	
Private school	215 (73.9%)	61 (28.4%)	7 (3.3%)	109 (50.7%)	38 (17.7%)	

Data are n (%) unless otherwise stated. Percentages in the Total column are column percentages of all 291 students; percentages in the Victim, Bully, Bully-victim, and Not involved columns are row percentages within each category. Numbers do not always sum to 291 because of missing responses

*F* Fisher’s exact test, *K* Kruskal–Wallis test

Perceived parenting style was not significantly associated with bullying roles in bivariate analysis (*p* = 0.099). Most participants reported an authoritative parenting style (74.2%). The victim role was most frequent among students reporting authoritarian parenting (38.1%, compared with 30.6% for authoritative parenting), and the bully-victim role was most frequent among those reporting uninvolved/neglectful (66.7%) or authoritarian (50.0%) parenting; students not involved in bullying were least frequent in the authoritarian group (9.5%). School type was significantly associated with bullying roles (*p* = 0.003). Private school participants were disproportionately represented among bully-victims, whereas public school participants were disproportionately represented among victims and bullies. Depressive symptoms differed strikingly across bullying roles (*p* < 0.001). Moderate-to-severe symptoms were reported by 61.7% of bully-victims and 42.0% of victims, compared with only 11.3% of students who were not involved. Involvement in cyber aggression was uncommon (5.2%) and was not significantly associated with bullying roles (*p* = 0.318).

### Patterns of bullying

In most cases, the bully was in the same class as the victim (41.6%), followed by the bully being in a different class within the same grade (19.9%). Bullying across grades was rare (Table 3). Regarding the perpetrators’ sex, most cases involved both boys and girls as perpetrators (31.6%). Among students who identified a single-sex perpetrator group, the most common patterns were girls bullied by boys (12.4%) and boys bullied by boys (10.7%). Bullying by a single perpetrator (32.0%) or by two to three perpetrators (28.2%) was the pattern most commonly reported. The most frequently reported duration was one to two weeks (25.8%), although 18.9% of the participants reported having been bullied for a year or longer. All percentages in this paragraph are of the whole sample (*N* = 291).

**Table 3:** Patterns of bullying, overall and by school type

Item	Overall (*N* = 291)	Public school (*n* = 76)	Private school (*n* = 215)	*p*-value
Class(es) of the bully				< 0.001 F
In my class	121 (41.6%)	17 (22.4%)	104 (48.4%)	
Different class, same grade	58 (19.9%)	18 (23.7%)	40 (18.6%)	
In a higher grade	8 (2.7%)	3 (3.9%)	5 (2.3%)	
In a lower grade	2 (0.7%)	0 (0.0%)	2 (0.9%)	
In different grades	18 (6.2%)	2 (2.6%)	16 (7.4%)	
Not bullied	81 (27.8%)	36 (47.4%)	45 (20.9%)	
Sex of perpetrators				< 0.001 F
Mainly by one girl	29 (10.0%)	4 (5.3%)	25 (11.6%)	
By several girls	15 (5.2%)	3 (3.9%)	12 (5.6%)	
Mainly by one boy	47 (16.2%)	15 (19.7%)	32 (14.9%)	
By several boys	21 (7.2%)	3 (3.9%)	18 (8.4%)	
By both boys and girls	92 (31.6%)	13 (17.1%)	79 (36.7%)	
Not bullied	82 (28.2%)	38 (50.0%)	44 (20.5%)	
Gender pattern of bullying				0.326 F
Boys bullied by boys	31 (10.7%)	10 (13.2%)	21 (9.8%)	
Boys bullied by girls	24 (8.2%)	5 (6.6%)	19 (8.8%)	
Girls bullied by boys	36 (12.4%)	8 (10.5%)	28 (13.0%)	
Girls bullied by girls	20 (6.9%)	2 (2.6%)	18 (8.4%)	
Number of perpetrators				< 0.001 F
Mainly one student	93 (32.0%)	19 (25.0%)	74 (34.4%)	
Two to three students	82 (28.2%)	12 (15.8%)	70 (32.6%)	
Four to nine students	20 (6.9%)	2 (2.6%)	18 (8.4%)	
More than nine students	5 (1.7%)	0 (0.0%)	5 (2.3%)	
Different students/grades	13 (4.5%)	5 (6.6%)	8 (3.7%)	
Not bullied	71 (24.4%)	36 (47.4%)	35 (16.3%)	
Duration of bullying				< 0.001 F
One or two weeks	75 (25.8%)	14 (18.4%)	61 (28.4%)	
About a month	45 (15.5%)	8 (10.5%)	37 (17.2%)	
About six months	9 (3.1%)	1 (1.3%)	8 (3.7%)	
About a year or more	55 (18.9%)	9 (11.8%)	46 (21.4%)	
Not bullied	97 (33.3%)	42 (55.3%)	55 (25.6%)	
Place of bullying (multiple responses)				
In class, with the teacher in the room	86 (29.6%)	18 (23.7%)	68 (31.6%)	0.247 C
In class, with the teacher not in the room	167 (57.4%)	24 (31.6%)	143 (66.5%)	< 0.001 C
Other places inside the school	176 (60.5%)	32 (42.1%)	144 (67.0%)	< 0.001 C
Outside the school	34 (11.7%)	8 (10.5%)	26 (12.1%)	0.875 C
Telling others about being bullied				< 0.001 C
Been bullied, told nobody	37 (12.7%)	5 (6.6%)	32 (14.9%)	
Been bullied, told somebody	170 (58.4%)	35 (46.1%)	135 (62.8%)	
Not bullied	67 (23.0%)	30 (39.5%)	37 (17.2%)	
Person told (multiple responses)				
Class teacher	62 (21.3%)	13 (17.1%)	49 (22.8%)	
Another adult at school	21 (7.2%)	7 (9.2%)	14 (6.5%)	
Parent(s)/guardian(s)	102 (35.1%)	18 (23.7%)	84 (39.1%)	
Brother(s)/sister(s)	81 (27.8%)	18 (23.7%)	63 (29.3%)	
Friend(s)	150 (51.5%)	30 (39.5%)	120 (55.8%)	
Somebody else	6 (2.1%)	1 (1.3%)	5 (2.3%)	
School liking				0.298 F
Do not or really do not like school	15 (5.2%)	3 (3.9%)	12 (5.6%)	
Neutral	131 (45.0%)	30 (39.5%)	101 (47.0%)	
Like or really like school	139 (47.8%)	43 (56.6%)	96 (44.7%)	
Number of friends in class				0.658 F
None	7 (2.4%)	1 (1.3%)	6 (2.8%)	
One to three	75 (25.8%)	22 (28.9%)	53 (24.7%)	
More than three	205 (70.4%)	52 (68.4%)	153 (71.2%)	
Afraid of being bullied				0.093 C
Never	94 (32.3%)	29 (38.2%)	65 (30.2%)	
Seldom to fairly often	142 (48.8%)	39 (51.3%)	103 (47.9%)	
Often to very often	49 (16.8%)	7 (9.2%)	42 (19.5%)	
Type of bullying, as victim or perpetrator (multiple responses)				
Physical	112 (38.5%)	23 (30.3%)	89 (41.4%)	0.115 C
Verbal: called mean names	162 (55.7%)	33 (43.4%)	129 (60.0%)	0.018 C
Verbal: threats	65 (22.3%)	11 (14.5%)	54 (25.1%)	0.079 C
Verbal: about race or colour	100 (34.4%)	22 (28.9%)	78 (36.3%)	0.309 C
Verbal: with sexual meaning	77 (26.5%)	13 (17.1%)	64 (29.8%)	0.046 C
Relational: excluded from the group	101 (34.7%)	17 (22.4%)	84 (39.1%)	0.013 C
Relational: rumours spread	84 (28.9%)	14 (18.4%)	70 (32.6%)	0.028 C
Damage to property	79 (27.1%)	13 (17.1%)	66 (30.7%)	0.032 C

Data are n (%). Percentages use *N* = 291 (overall), *n* = 76 (public school), and *n* = 215 (private school) as denominators. Place of bullying, person told, and type of bullying items allowed multiple responses, so percentages do not sum to 100%. A “Not bullied” row is shown for each block describing the experience of being bullied, so that the categories displayed account for the whole sample apart from missing responses and each p-value refers to the categories shown. The number of students in the “Not bullied” row differs between blocks because each block derives from a separate questionnaire item with its own pattern of missing responses. Gender-pattern rows include only students who reported being bullied by a single-sex perpetrator group; students bullied by both boys and girls appear in the “Sex of perpetrators” block. Type-of-bullying rows count students who reported that form of bullying either as a victim or as a perpetrator at two or three times a month or more

*F* Fisher’s exact test, *C* Chi-squared test

Bullying most often occurred in unsupervised areas inside the school (e.g., playgrounds, hallways, and toilets) (60.5%) and in classrooms when teachers were absent (57.4%). Just over half of the participants (58.4%) reported having been bullied and having told someone about it, most commonly a friend (51.6%), followed by parents (35.1%) and teachers (21.3%). The most common form of bullying was verbal name-calling (55.7%), followed by physical bullying (38.5%), social exclusion (34.7%), racial- or colour-based bullying (34.4%), and rumour spreading (28.9%). Several forms of bullying, including name-calling, social exclusion, rumour spreading, property damage, and sexually explicit comments, were reported significantly more often by private school participants than by public school ones, whereas the differences for physical bullying, threats, and race- or colour-based comments did not reach statistical significance.

### Adult communication and bystander behaviour

A substantial proportion of participants reported that teachers (23%) and guardians (24%) had never discussed bullying with them (Table 4). Only 8% of the guardians frequently discussed bullying. Regarding the degree of action taken by teachers when bullying was identified, 28% of the participants felt that teachers acted somewhat, 35% reported that teachers acted quite a lot or a lot, and 21% believed that teachers did very little or nothing. A small proportion of participants (8%) said they would join in bullying a classmate they disliked. When witnessing their peers being bullied, 36% of the participants reported that they attempted to help, 26% felt that they should have helped but did nothing, 9% considered bullying normal and did not intervene, and 2% joined in bullying (Table 4).

**Table 4:** Teacher and guardian communication about bullying and bystander responses (*N* = 291)

Item	*n* (%)
Teacher(s) have talked with the student about the student bullying others	
I have not bullied other students	154 (52.9%)
Never talked about it	67 (23.0%)
Talked about it once	38 (13.1%)
Talked about it several times	24 (8.2%)
Missing	8 (2.7%)
Anyone at home has talked with the student about the student bullying others	
I have not bullied other students	153 (52.6%)
Never talked about it	71 (24.4%)
Talked about it once	39 (13.4%)
Talked about it several times	22 (7.6%)
Missing	6 (2.1%)
Perceived teacher action to counteract bullying	
Very little or nothing at all	62 (21.3%)
Quite little	31 (10.7%)
Somewhat	82 (28.2%)
Quite a lot	52 (17.9%)
A lot	50 (17.2%)
Missing	14 (4.8%)
Would join in bullying a student they dislike	
Yes	23 (7.9%)
Maybe	48 (16.5%)
I do not know	62 (21.3%)
Probably not	26 (8.9%)
No	47 (16.2%)
Definitely not	81 (27.8%)
Missing	4 (1.4%)
Reaction when seeing a peer being bullied	
Never noticed bullying	62 (21.3%)
Joined in the bullying	6 (2.1%)
Did nothing, considering bullying to be normal	25 (8.6%)
Just watched	11 (3.8%)
Did nothing, but felt they should help the victim	75 (25.8%)
Tried to help the victim	104 (35.7%)
Missing	8 (2.7%)

Data are n (%) of all 291 students. Derived from R-OBVQ items on communication with adults about bullying and bystander behaviour

### Independent factors associated with bullying role

In the multinomial logistic regression model (Table 5), several factors were independently associated with bullying roles after adjusting for sex, sex–gender alignment, age, height-for-age, weight-for-height, school type, and perceived parenting style. Studying in a private school was associated with a higher relative risk of being a bully-victim (RRR 4.48, 95% CI 1.97–10.22; *p* < 0.001) but was not associated with being a victim or a bully. Compared with authoritative parenting, authoritarian parenting was associated with a higher relative risk of being a bully-victim (RRR 4.20, 95% CI 1.24–14.21; *p* = 0.021); a comparable but non-significant trend was observed for the victim role (RRR 2.77, 95% CI 0.81–9.49; *p* = 0.105). Shorter-than-average stature for one’s age was associated with a lower relative risk of being a bully-victim (RRR 0.27, 95% CI 0.08–0.93; *p* = 0.039). Larger-than-average body size for one’s height showed a positive but non-significant association with being a bully-victim (RRR 2.32, 95% CI 0.82–6.57; *p* = 0.114) and a similar trend for the victim role (RRR 2.63, 95% CI 0.90–7.65; *p* = 0.077). Estimates for the bully-only role were unstable owing to the small number of students in this group, and two body-size categories could not be estimated because of quasi-complete separation; these estimates should be interpreted with caution. Sex, sex–gender alignment, and age were not significantly associated with any bullying role. The overall model performed significantly better than the null model (likelihood-ratio χ² = 59.2, df = 30, *p* = 0.001; McFadden’s pseudo-R² = 0.093; AIC = 639.9).

**Table 5:** Multinomial logistic regression analysis of factors associated with bullying role (reference category: not involved)

Factors (reference)	Victim		Bully		Bully-victim	
	RRR (95% CI)	*p*-value	RRR (95% CI)	*p*-value	RRR (95% CI)	*p*-value
Sex–gender alignment: not aligned (aligned)	1.48 (0.25, 8.70)	0.663	4.37 (0.48, 39.99)	0.191	2.47 (0.49, 12.55)	0.275
Female sex (male)	1.20 (0.58, 2.47)	0.630	2.41 (0.45, 12.94)	0.305	0.90 (0.45, 1.81)	0.777
Age (per additional year)	0.79 (0.46, 1.33)	0.372	0.59 (0.21, 1.66)	0.318	1.10 (0.66, 1.83)	0.708
Private school (public school)	1.59 (0.72, 3.53)	0.251	0.89 (0.21, 3.84)	0.876	4.48 (1.97, 10.22)	< 0.001 *
Parenting style (authoritative)						
Authoritarian	2.77 (0.81, 9.49)	0.105	1.84 (0.17, 20.36)	0.620	4.20 (1.24, 14.21)	0.021 *
Permissive or uninvolved/neglectful	0.60 (0.14, 2.50)	0.483	3.08 (0.53, 17.98)	0.212	1.33 (0.40, 4.41)	0.646
Height for age (average)						
Shorter than average	0.48 (0.16, 1.45)	0.194	NE	—	0.27 (0.08, 0.93)	0.039 *
Taller than average	0.57 (0.15, 2.15)	0.405	1.11 (0.11, 11.30)	0.933	1.74 (0.58, 5.21)	0.325
Weight for height (well proportioned)						
Thinner than average	1.67 (0.66, 4.21)	0.276	0.84 (0.15, 4.88)	0.848	0.84 (0.32, 2.20)	0.724
Larger than average	2.63 (0.90, 7.65)	0.077	NE	—	2.32 (0.82, 6.57)	0.114

*RRR* Relative-risk ratio, *CI* Confidence interval. *p < 0.05. Model fit: likelihood-ratio test versus the null model, χ² = 59.2, df = 30, *p* = 0.001; McFadden’s pseudo-R² = 0.093; AIC = 639.9. Estimates are from the re-specified multinomial model (sex entered a priori; religion and grade-point average excluded); reference category, not involved; complete-case *N* = 270. NE = not estimable owing to quasi-complete separation (only 11 students were classified as bully-only)

## Discussion

Among the 291 Grade 5 and Grade 6 students from one public and one private school in Hat Yai, southern Thailand, the prevalence of bullying involvement was 78.7%, and the most common role was that of a bully-victim (44.7%). This high figure partly reflects the inclusive definition used in this study (having bullied others or been bullied at least two or three times a month on any of the R-OBVQ victimisation or perpetration items), which favours sensitivity over specificity and captures lower-intensity bullying that stricter weekly definitions would have excluded. Restricting the classification to the two global R-OBVQ items lowered the involvement prevalence to 59.1%, which illustrates how strongly the case definition drives the estimate. Several additional factors may have contributed. Methodologically, anonymous self-administered questionnaires completed without teachers present tend to elicit fuller disclosure than teacher or parent reports, and our four-role classification counted any qualifying involvement, whether as victim, bully, or both. Sample-related factors are also plausible: only 291 of the 429 eligible students participated (67.8%), and if families with greater concern about bullying were more willing to consent, the estimate would be inflated; conversely, affected families may also have declined, so the direction of any participation bias is uncertain. Contextually, both schools were large urban institutions, and almost three-quarters of the participants attended the private school, in which bully-victim status and most specific forms of bullying were more common; the pooled estimate therefore weights the higher-prevalence school more heavily. Culturally, teasing and mocking are often framed as playful interaction in Thai society, which may normalise low-level peer aggression and delay recognition and adult intervention [20]. Our estimate should also be viewed alongside other studies of primary school students. Substantial levels of bullying involvement have been reported among rural Indian primary school students [29], among elementary school children across seven European countries [30], and among primary and lower-secondary students in Pattani province in southern Thailand [31, 32]. Most of these estimates are nevertheless lower than ours. Our estimate of 78.7% is lower than the 91.8% reported in the 2020 Thai national report [19] and considerably higher than the approximately 32% global average estimated by UNESCO [18]. Taken together, the very high prevalence observed here is consistent with the documented severity of bullying in Thailand [17–19]. The dominance of the bully-victim role likewise mirrors the findings of several Asian and European school-based studies [29, 30, 33–35]. Nevertheless, the absolute figure should be interpreted cautiously in light of the inclusive case definition, the two-school urban sample, and the modest participation rate, and it should not be generalised to Thai primary schools at large.

From the perspective of child and adolescent mental health, this is of particular concern because, compared with victims and bullies, bully-victims consistently exhibit the poorest psychological and psychiatric outcomes, including higher depression, anxiety, suicidality, and long-term internalising and externalising problems [4–10, 14, 15].

### Parenting style and family environment

Authoritarian parenting, characterised by strict control and limited emotional warmth, was associated with a higher relative risk of being a bully-victim, with a comparable but non-significant trend for the victim role. The overall bivariate association between parenting style and bullying role did not reach statistical significance (*p* = 0.099), so this finding rests on the adjusted model and should be interpreted accordingly. This finding is consistent with longitudinal evidence that harsh, low-warmth parenting environments contribute to children’s vulnerability to victimisation and to their own use of aggression [5, 34]. Furthermore, the finding presents salient implications for clinical practice. In Thai primary care and child mental health settings, brief screening for parenting style during well-child or behavioural health visits may help identify children at heightened risk of bullying involvement, and interventions aimed at parenting skills may serve as an adjunct to school-based prevention.

### Body size and physical characteristics

In the multivariable model, a larger body size for one’s height showed a positive but non-significant trend toward a higher relative risk of being a bully-victim, whereas a shorter stature for one’s age was independently associated with a lower relative risk of being a bully-victim. The direction of the first association is consistent with international evidence that overweight and obese children are at increased risk of peer victimisation and of bullying involvement as both the target and the perpetrator [8, 31, 36–38]. In the present sample, however, this association did not reach statistical significance and should be regarded as hypothesis-generating. The protective association observed for shorter stature is less commonly reported and may reflect either lower social visibility or a culture-specific dynamic in southern Thailand; therefore, replication in larger samples is warranted. From a clinical standpoint, this context-specific pattern underscores that the relationship between body size and bullying involvement may vary by setting. Primary care and school health practitioners in southern Thailand should screen for bullying involvement on an individual basis rather than presume that a smaller stature confers greater vulnerability. Because height and weight were self-reported rather than measured, both body-size findings should be regarded as provisional.

### Mental health: the bully-victim burden

Moderate-to-severe depressive symptoms were reported by 61.7% of bully-victims and 42.0% of victims. These estimates were in marked contrast to the percentage observed among participants not involved in bullying (11.3%). Although the cross-sectional design of this study precludes causal inference, depressive symptoms are most plausibly a consequence rather than a determinant of bullying involvement. Thus, the PHQ-A score was not entered as a predictor in the regression analysis, so as to avoid reverse causation and over-adjustment. Nevertheless, the strength of the association underscores the need to integrate routine mental health screening with bullying-prevention measures. Care pathways should ensure that bullied students, particularly bully-victims, have low-threshold access to school counsellors and community child-psychiatry services [9, 13, 35, 39].

### School type and environment

Private school students were significantly more likely to be bully-victims than public school students, and many forms of bullying (name-calling, social exclusion, rumour spreading, property damage, and sexual comments) were more common in private schools. Differences in classroom size, peer-group stability, supervision intensity, and disciplinary culture between Thai public and private schools are plausible mediators of this pattern. However, because school type is closely entangled with family socioeconomic status, class size, and students’ willingness to disclose bullying, this strong association should be interpreted with caution and not as causal. It may partly reflect residual confounding or unmeasured contextual differences between public and private schools rather than a direct effect of the private school setting [40, 41]. Bullying overwhelmingly occurred in low-supervision spaces, such as playgrounds, hallways, toilets, and unattended classrooms [31]. Improving adult presence in these spaces is a low-cost, high-impact prevention measure.

### Reporting and adult response

Although most bullied participants had told someone about it, only one in five of all participants had told a teacher (21.3%). Additionally, nearly a quarter of the participants reported that teachers and guardians had never discussed bullying with them. This communication gap is consistent with the findings of prior Thai studies [31, 32, 42] and likely contributes to the under-recognition of bullying in primary care and school health services. Students’ responses to witnessing bullying were similarly mixed: while 36% of the participants reported attempting to help, more than a quarter felt that they should have helped but did not. This pattern suggests that classroom-level bystander-empowerment programmes (such as KiVa-style interventions) may represent a valuable addition to bullying-prevention efforts in Thailand.

### Strengths and limitations

The strengths of this study include the use of validated, role-discriminating instruments (the Thai R-OBVQ, CAPVS, and PHQ-A) and the inclusion of both public and private school students. Additionally, this study assessed both traditional bullying and cyberbullying and classified participants by their role in bullying (not involved, victim, bully, and bully-victim). This enabled the particularly vulnerable bully-victim subgroup to be identified rather than obscured within broader victim or bully categories. The study also helps to address an evidence gap by focusing on younger primary school children in Grades 5 and 6 in southern Thailand, an age group and region under-represented in previous bullying research. All enrolled Grade 5 and 6 students at both schools were invited, as a complete grade-level census.

However, several limitations warrant emphasis. First, the sample was drawn from two schools in a single district and may not fully represent southern Thailand or the country. The two schools were purposively selected to represent the two dominant types serving the urban Hat Yai population, one large public and one large private institution, thereby capturing a socioeconomically varied cross-section of children. Nevertheless, rural schools and other provinces were not sampled, so generalisation beyond comparable urban settings should be made cautiously. In addition, the achieved sample of 291 students fell short of the calculated requirement of 426 and represented 67.8% of those eligible, so the study was somewhat underpowered and participation bias cannot be excluded; as discussed above, the direction of any such bias is uncertain. Second, all measures were self-reported and may be subject to social desirability and recall bias, although the questionnaires were anonymous and administered without teachers present. Height and weight in particular were reported by the students rather than measured, so the height-for-age and weight-for-height categories are subject to misclassification. Children of this age tend to over-report height and under-report weight, and such misclassification is likely to have attenuated rather than created the body-size associations reported here. Third, the cross-sectional design precludes causal inference; longitudinal research is needed to clarify the temporal relationships among parenting environment, bullying involvement, and depressive symptoms. Fourth, the small number of students classified as bullies (*n* = 11) limited statistical power and produced wide confidence intervals; the estimates for this role should therefore be regarded as exploratory and interpreted with caution. Fifth, perceived parenting style was assessed using a single investigator-compiled item; although based on an established typology [22], a single item cannot capture the full complexity of parenting behaviour, and some misclassification is possible. Sixth, the prevalence of cyber aggression in this primary school sample was very low and may have been underestimated. This likely reflects the limited independent access to smartphones and social media at this age, closer parental monitoring of children’s online activity, possible under-reporting, and the limited sensitivity of the CAPVS in younger children. Replication in older students with greater device access is warranted.

## Conclusions

In this cross-sectional study of Grade 5 and 6 students in Hat Yai, southern Thailand, school bullying involvement was highly prevalent, and bully-victims formed the largest and most psychologically burdened group. Attending a private school and authoritarian parenting were independently associated with a higher relative risk of being a bully-victim, whereas a shorter stature was associated with a lower relative risk. The very strong association between moderate-to-severe depressive symptoms and being a bully-victim emphasises that primary school bullying is not only a school-discipline issue; rather, it is fundamentally a child and adolescent mental health issue. Multilevel prevention, combining parenting-skills interventions, mental health screening in primary care and school clinics, supervision of low-visibility school spaces, and bystander-empowerment programmes, should be considered in Thai primary school settings.

## Data Availability

The de-identified datasets generated and analysed during the current study are available from the corresponding author on reasonable request.

## Abbreviations

AIC: Akaike information criterion
CAPVS: Cyber-Aggression Perpetration/Victimization Scale
CI: Confidence interval
GPA: Grade-point average
IQR: Interquartile range
PHQ-A: Patient Health Questionnaire for Adolescents
R-OBVQ: Revised Olweus Bully/Victim Questionnaire
RRR: Relative-risk ratio
UNESCO: United Nations Educational, Scientific and Cultural Organization
